# Supplementary material for: Development of Solvent-Free Co-Ground Method to Produce Terbinafine Hydrochloride Cyclodextrin Binary Systems; Structural and In Vitro Characterizations
Source: Pharmaceutics. 2022 Mar 30;14(4):744. doi: 10.3390/pharmaceutics14040744 (PMC9025016; doi:10.3390/pharmaceutics14040744)
Supplement: Supplementary file 1 [file pharmaceutics-14-00744-s001.zip › pharmaceutics-1620961-supplementary.pdf]

pharmaceutics

Supplementary

Development of Solvent-free Co-ground Method to Produce Terbinafine Hydrochloride Cyclodextrin Binary Systems; Structural and In Vitro Characterizations

Balázs Attila Kondoros, Orsolya Jójárt-Laczkovich, Ottó Berkesi, Piroska Szabó-Révész, Ildikó Csóka, Rita Ambrus and Zoltán Aigner

Table S1. contains the dissolution efficiency (DE, %) at 120 min and MDT values. DE of physical mixtures (PMs) is higher than pure drug, due to the solubilizing effect of dissolved CDs, however lower than ground products.

Table S1. Calculated dissolution efficiency (DE, %) at 120 min and MDT values of pure drug, PMs and co-ground products.

	Products	% DE 120 min	MDT
Simulated intestinal medium	TER	0.90	18.88
	TER:DIMEB_PM	4.11	12.84
	TER:HPBCD_PM	1.27	12.12
	TER:DIMEB	4.81	11.02
	TER:HPBCD	1.70	28.86
Simulated gastric medium	TER	80.45	18.35
	TER:DIMEB_PM	74.91	9.13
	TER:HPBCD_PM	84.87	10.43
	TER:DIMEB	91.54	12.54
	TER:HPBCD	88.54	7.69

Table S2. shows the calculated apparent permeability coefficient (Papp) and enhancement ratios (R). In case of DIMEB-products, co-ground products and PMs showed similar permeability. However, diffusion from HPBCD containing PMs decreased from intestinal medium, the diffused drug was undetectable under the measurement conditions. On the other hand, the drug diffused from gastric medium resulted in a higher concentration in the acceptor phase than the ground products.

Table S2. Calculated apparent permeability coefficient (P_{app}) and enhancement ratios (R) of pure drug, physical mixtures (PM) and co-ground products.

	Products	P_{app}	R
Simulated intestinal medium	TER	1.41E-04	-
	TER:DIMEB_PM	1.34E-04	0.950
	TER:HPBCD_PM	-	-
	TER:DIMEB	1.30E-04	0.921
	TER:HPBCD	1.12E-04	0.793
Simulated gastric medium	TER	7.72E-06	-
	TER:DIMEB_PM	1.76E-06	2.280
	TER:HPBCD_PM	1.86E-06	2.422
	TER:DIMEB	1.72E-04	2.232
	TER:HPBCD	3.28E-05	0.425
